# Efficient Vapor‐Phase Selective Hydrogenolysis of Bio‐Levulinic Acid to γ‐Valerolactone Using Cu Supported on Hydrotalcite Catalysts

**DOI:** 10.1002/gch2.201800028

**Published:** 2018-07-11

**Authors:** Harisekhar Mitta, Prem Kumar Seelam, K. V. Raghava Chary, Suresh Mutyala, Rajender Boddula, Inamuddin, Abdullah M. Asiri

**Affiliations:** ^1^ State Key Laboratory of Catalysis Dalian Institute of Chemical Physics Chinese Academy of Sciences Dalian 116023 China; ^2^ Catalysis Division CSIR—Indian Institute of Chemical Technology Hyderabad 500007 Telangana India; ^3^ Environmental and Chemical Engineering Unit Faculty of Technology University of Oulu P.O. Box 4300 FI‐90014 Oulu Finland; ^4^ CAS Key Laboratory of Nanosystem and Hierarchical Fabrication National Centre for Nanoscience and Technology No. 11 ZhongGuanCun, BeiYiTiao 100190 Beijing P. R. China; ^5^ Chemistry Department Faculty of Science King Abdulaziz University Jeddah 21589 Saudi Arabia; ^6^ Centre of Excellence for Advanced Materials Research King Abdulaziz University Jeddah 21589 Saudi Arabia

**Keywords:** bio‐levulinic acid, catalysts, hydrogenolysis, hydrotalcite, γ‐valerolactone

## Abstract

In this work, Cu nanoparticles (Cu NPs, 2‐20 nm) supported on Hydrotalcite catalysts exhibit enhanced selectivity for γ‐valerolactone (GVL) during hydrogenolysis of levulinic acid (LA). At 260 °C, over 3 wt% Cu achieved 87.5% of LA conversion with a maximum GVL selectivity (95%). In contrast, LA hydrogenolysis over 3Cu/Hydrotalcite catalyst is highly active and stable toward the production of GVL due to balanced acido‐basicity and higher Cu dispersion with ultrasmall particle sizes, which are investigated through the temperature programmed desorption (TPD) of ammonia, N_2_O titration, and transmission electron microscopy (TEM) analysis. Hydrotalcite in combination with inexpensive Cu catalyst is found to be an efficient and environmentally benign for LA hydrogenolysis.

## 1 Introduction

In the future, given the anticipated technological advancements in finding and extracting fossil‐based fuels will be expensive and environmentally unfriendly. Moreover, the nonconventional energy resources such as biomass and other renewables sources are competitive with fossil‐based feedstocks. Currently, plenty of research focused on to produce clean and renewable fuels and chemicals, and also to find out the most economical and greener processes which could replace the current unstainable processes.^1^, ^2^ The research activities are mainly concentrated on catalytic transformation of different biomass‐derived compounds, which are more easily accessible, renewable, and environmentally benign.^3^, ^4^ In these lignocellulosic biomass‐based chemicals is levulinic acid (LA) which is found to be a sustainable feedstock for biorefinery to produce high value‐added chemicals and intermediates.^5^, ^6^

LA is one of the building blocks and also part of polyols platform for biorefinery concept and being a by‐product of the biomass processing unit. Further, it can be converted to a variety of chemicals, fuels, and intermediates. Among several transformations, the hydrogenolysis of LA to γ‐valerolactone (GVL) is very interesting and a green synthesis route. Moreover, the GVL is an important platform molecule and used in many areas including as a green solvent,^7^ an intermediate to produce renewable fuels and chemicals.^8^, ^9^ The development of environmentally benign and a cost‐efficient process for the synthesis of GVL has received attention. Recently, production of GVL has been developed via several routes using different catalysts and hydrogen sources for the reduction of LA.^10^, ^11^ Conventionally, GVL can be produced through chemical transformation either over homogeneous or heterogeneous catalysts at low temperatures.^12^, ^13^

Generally, catalytic vapor‐phase hydrogenolysis of LA was performed over different heterogeneous catalysts such as noble metal catalysts (Ru, Pd, and Au on different supports or carrier materials) and achieved moderately higher LA conversions and GVL selectivity.^14^, ^15^ In addition, transition base metals such as Cu and Ni are also found to be active and exhibit similar activities as that of noble metal catalysts, and, moreover, base metals are inexpensive compared to noble metals, but possess few drawbacks (e.g., deactivation). Recently, several research groups demonstrated over vapor‐phase LA hydrogenolysis using non‐noble metal–supported catalysts.^16^, ^17^ Among the base metal, copper‐based catalysts have been reported high activity in producing GVL selectively from LA hydrogenolysis. Cu‐based catalysts either in bulk or as supported forms have shown good catalytic performance in LA hydrogenolysis.^12^ An overview of the performance of various Cu‐based supported catalysts in a conventional fixed‐bed reactor are summarized in **Table** **1**.^18^, ^19^, ^20^, ^21^ The LA hydrogenolysis reaction takes place over bifunctional metal‐supported catalysts under H_2_ flow and, thus, exhibits high activity.

**Table 1:** Comparison of various Cu‐supported catalysts published in literature with different reaction conditions using vapor‐phase LA hydrogenolysis

Catalyst	H_2_ source	Reaction conditions				Catalytic activity		Ref.
		*T* [°C]	*P* [MPa]	Time [h]	WHSV [h^−1^]	LA Conv. [%]	GVL Sel. [%]	
5 wt% Cu/SiO_2_	H_2_	265	1.0	100	0.513	100	99.9	18
20.5 wt% Cu/SiO_2_	H_2_	240	3.0	100	0.3	95	93.8	19
20.3 wt%Cu/SiO_2_–Al_2_O_3_	H_2_	240	3.0	100	0.3	99.8	95.8	19
5 wt% Cu/ZrO_2_	H_2_	265	0.1	20	0.169	81	83	20
(2:1) Cu–Zn Al_2_O_3_	H_2_	240	0.1	30	1.71	96.4	94	21
20 wt% Cu/Al_2_O_3_	H_2_	240	0.1	5	1.7	93.7	91.5	12
3Cu/Hydrotalcite	H_2_	265	0.1	23	0.456	87.5	95	This study

Mg–Al Hydrotalcite (Hydrotalcite, HT) is an anionic bifunctional, mixed metal oxides, and there are positively charged cations (metal ions) balanced with charged anions (OH^−^ and CO_2_^−2^) and water molecules located in the interlayer region.^22^, ^23^ These materials are found to be more attractive and novel due to their unique properties such as acidity, tunability, and possess moderately high surface area.^24^, ^25^ Moreover, the materials' properties depend on the nature of the cations' incorporation into Hydrotalcite interlayered carbonate anions. Jablonska et al.^26^ and Wang et al.^27^ reported that the acidic properties of the Hydrotalcite mixed oxides play an important role in many catalytic applications like aldol condensation, transesterification, dehydrogenation, and redox reactions. Interestingly, the Hydrotalcite structure recovers easily when the material is exposed to water by incorporating OH^−^ as charge‐balancing anions as well as irreversible transformation can occur into an interdispersed mixed oxide; thus, this material can be applied for different reactions due to these unique properties. Thus, the Hydrotalcite‐type materials and their derivatives have been widely investigated due to remarkable physicochemical properties.

In this work, we studied a novel approach of the colloidal deposition of highly dispersed Cu nanoparticles (NPs) over Hydrotalcite using ethanol as solvent. Detailed catalysts' characterizations were analyzed using different techniques reported in this work. Herein, specifically, we examine tuning Cu loading, the size effect of copper NPs, metal dispersion, and acidic properties, as well as their catalytic performance for producing GVL in a fixed bed stainless steel reactor under 0.1 MPa H_2_ pressure.

## 2 Results and Discussion

### 2.1 Structural Study

**Figure 1:**
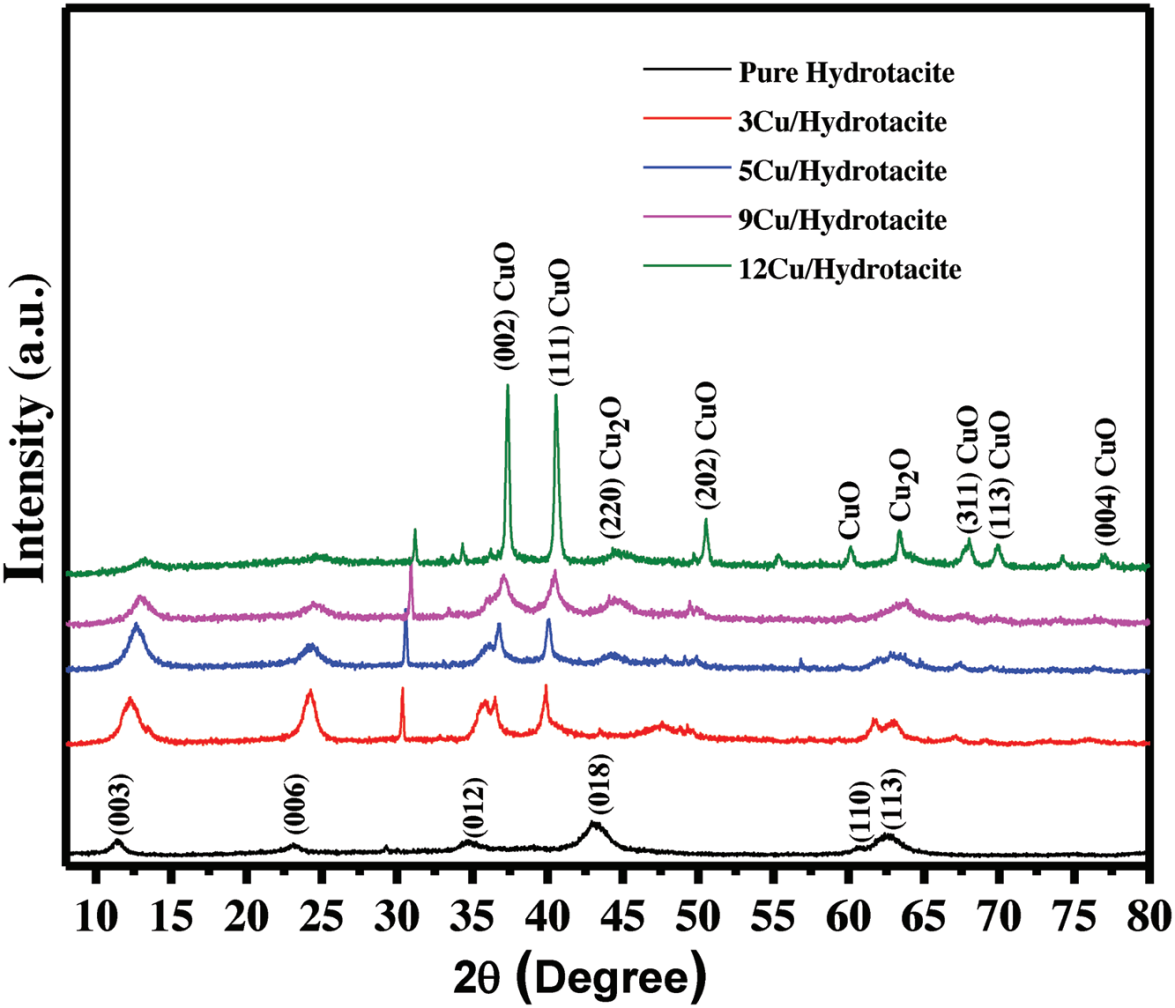
XRD patterns of pure Hydrotalcite and various Cu/Hydrotalcite‐based catalysts.

The X‐ray powder diffraction (XRD) patterns of pure Hydrotalcite and various Cu‐loaded Hydrotalcite catalysts are shown in **Figure** **1**. The pure Hydrotalcite showed sharp diffraction peaks at 2θ = 11.4°, 23.2°, 60.3°, and 62.7°, which represent the crystalline planes of (003), (006), (110), and (111), respectively, which attributes the formation of Hydrotalcite phases in the layered structure (according to JCPDS NO. 41‐1428). Additionally, a strong broad and symmetric reflection is also appeared at 2θ = 35.1° and 43.1°; this confirms the characteristic features of well‐crystallized Hydrotalcite (JCPDS 41‐1428). Therefore, this confirms that the most of Mg^+2^ ions in brucite‐like layers were successfully replaced by Cu^+2^ ions.

Kannan et al.^28^ have reported a similar phenomenon on diffraction patterns of Cu‐based Hydrotalcite catalysts. Furthermore, the diffraction patterns of various fresh Cu‐supported Hydrotalcite catalysts were shown in Figure 1. The absence of Cu_2_O and the minor CuO peaks at 35.6°, 38.8° was noticed for 3 wt% Cu catalyst. In the Cu loadings above 3 wt%, the diffraction peaks of Cu crystalline phases appeared at 2θ = 35.6°, 38.8°, 48.7°, 53.5°, 58.3°, 61.5°, and 66.2°, which are assigned to crystalline planes of (002), (111), (202), (311), (113), and (004), respectively(JCPDS.41‐254).^25^, ^28^ In addition, the other three Cu_2_O peaks at 29.60 °, 42.6 °, and 61.07 ° diffraction angles were assigned to the (110), (200), and (220) crystal planes (JCPDS No. 65‐3288). As expected, the peak intensities of CuO and Cu_2_O reflections were found to increase with Cu loadings. Similarly, the XRD results also confirmed that the Cu_2_O/CuO phases were disappeared and were difficult to analyze for 3 wt% Cu catalyst due to low loading. In the case of higher loadings, i.e., above 3 wt%, the crystalline phases of CuO/Cu_2_O were appeared and, thus, formed bigger particle sizes (confirmed in **Table** **2**).The average Cu crystallite size was calculated by using the Debye–Scherrer equation, and the Cu size increases from 2.1 to 17.2 nm as the loading increases from 3 to 15 wt% catalysts (Table 2).

**Table 2:** Physico‐chemical properties of Hydrotalcite support and Cu‐loaded/Hydrotalcite catalysts

Texture properties				N_2_O decomposition			XRD
Cu [wt%]	BET‐surface area) [m^2^ g^−1^]	Pore‐diametera) [nm]	Pore volume a) [cc g^−1^]	Metal dispersion b) [%]	Metal areab) [m^2^ g^−1^]	ICP Cu [wt%]	Crystalline sizec) [nm]
0.0	119	27.8	0.539	0	0	0	0
3	92	30.2	0.040	40	110	1.9	2.1
5	69	31.7	0.101	23	211	3.8	5.7
9	51	28.3	0.062	15	285	7.1	9.3
12	36	23.4	0.051	10	342	9.8	17.2

^a)^ Surface area, pore diameter, and pore volume measured from nitrogen adsorption–desorption isotherm

^b)^ Metal dispersion and metal area calculated by the N_2_O decomposition

^c)^ Crystallite size calculated by the XRD analysis.

**Figure 2:**
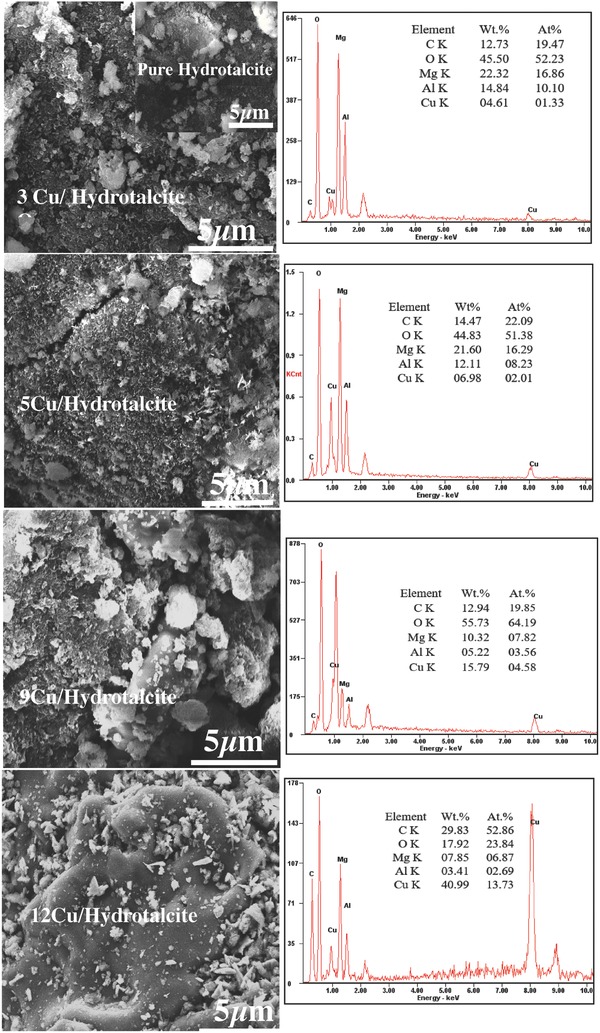
SEM images of pure Hydrotalcite and various Cu‐loaded Hydrotalcite catalysts.

Surface composition and morphological images of all catalysts were determined by SEM–EDS and are represented in **Figure** **2**. The image magnification of 15 000 times was fixed for all the catalysts during scanning electron microscopy (SEM) analysis. The presence of Cu, Al, Mg, and O elements was detected from energy dispersive spectrometer (EDS), and this represents the surface composition of the catalysts; the presence of surface was also confirmed by SEM analysis. In Figure 2, the microscopic images showed that the parent Hydrotalcite support has a sheet‐type structure. The amount of Cu (in wt%) was also determined by inductively coupled plasma (ICP) analysis and the values are presented in Table 2. The Cu content was found to be lower than the nominal loadings, and this deviation from the target loadings was due to the Cu leaching that occurs during the preparation steps.

**Figure 3:**
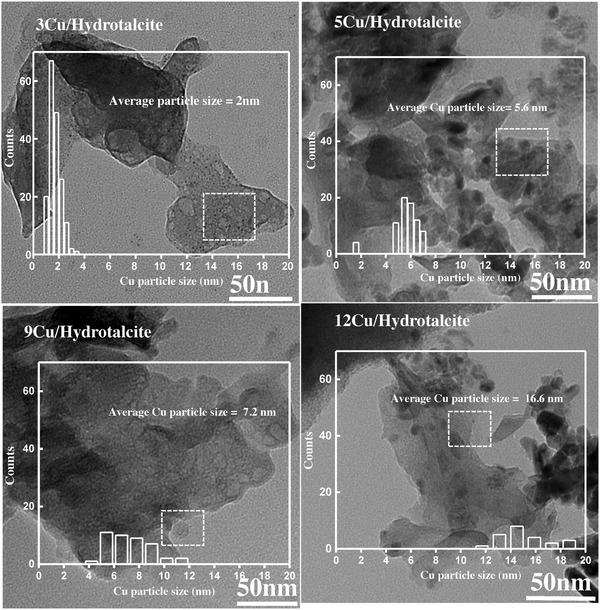
TEM images and amount of Cu particles' distribution of various Hydrotalcite‐based Cu catalysts.

The transmission electron microscopy (TEM) images and particle size distribution of all Cu‐supported catalysts are shown in **Figure** **3**. The average Cu particle size of all catalysts was found to be in the range from ≈2 to ≈16.6 nm. Over 3Cu/Hydrotalcite catalyst, an ≈2 nm particle was found that displayed a narrow and highly homogeneous distribution over the Hydrotalcite surface. The average Cu particle size situated in the range of 5.6–16.6 nm at higher loadings, i.e., above 3 wt%, and this larger particle sizes are due to the Cu particles' agglomeration. Thus, it clearly indicates that high dispersion with smaller Cu particle size occurs significantly at lower Cu loadings.

**Figure 4:**
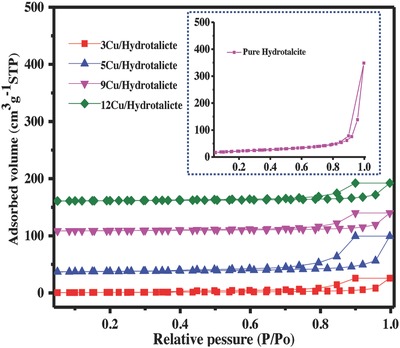
N_2_ adsorption–desorption isotherms for pure Hydrotalcite support (inset) and various Cu‐loaded Hydrotalcite catalysts.

Specific Brunauer‐Emmett‐Teller (BET) surface area, pore volume, and pore size were calculated from the N_2_ physisorption isotherm measurements, and the textural properties are presented in Table 2. The isotherm patterns of pure Hydrotalcite and various Cu‐based catalysts are shown in **Figure** **4**. All catalysts exhibited type IV isotherms with a hysteresis loop caused by capillary condensation, indicating the characteristic of mesoporous nature of the catalysts with the relatively lower specific surface area (according to International Union of Pure and Applied Chemistry (IUPAC) classification). Similar approach was used by Pavel et al.^29^ and Wang and Jehng^23^ over transition metal–loaded Hydrotalcite‐based catalytic materials. The surface area, pore volume, and diameter of pure Hydrotalcite support are 119 m^2^ g^−1^, 0.539 cc g^−1^, and 27.8 nm, respectively. After Cu impregnation, the surface areas of the catalysts declined from 119 to 36 m^2^ g^−1^, and, moreover, the pore volume and diameter for all the catalysts in the range of 0.040–0.051 cc g^−1^ and 30.2–23.4 nm, which are lower than the pure support. The decreasing trend is due to the blockage of pores by Cu particles. A similar trend was observed in the case of SBA‐15‐supported copper catalysts.^30^

The results of Cu dispersion, metal area, and particle size were measured by N_2_O decomposition, and the values are presented in Table 2. It was observed that the highest Cu dispersion and metal area were obtained in the 3 wt% Cu catalysts due to ultrafine smaller Cu particles' coverage over the entire surface. The Cu dispersion decreases with the Cu loadings due to the formation of bigger particles, i.e., the particle agglomeration on the catalyst surface occurs at higher loadings. Henceforth, this phenomenon was also observed by TEM and XRD analysis. Overall, the decline in Cu dispersion, Cu metal area, and the increment in average particle size were correlated with Cu loadings (Table 2). Jiang et al.^31^ and Mitta et al.^32^ observed similar phenomena on copper incorporated into various supported catalysts.

**Figure 5:**
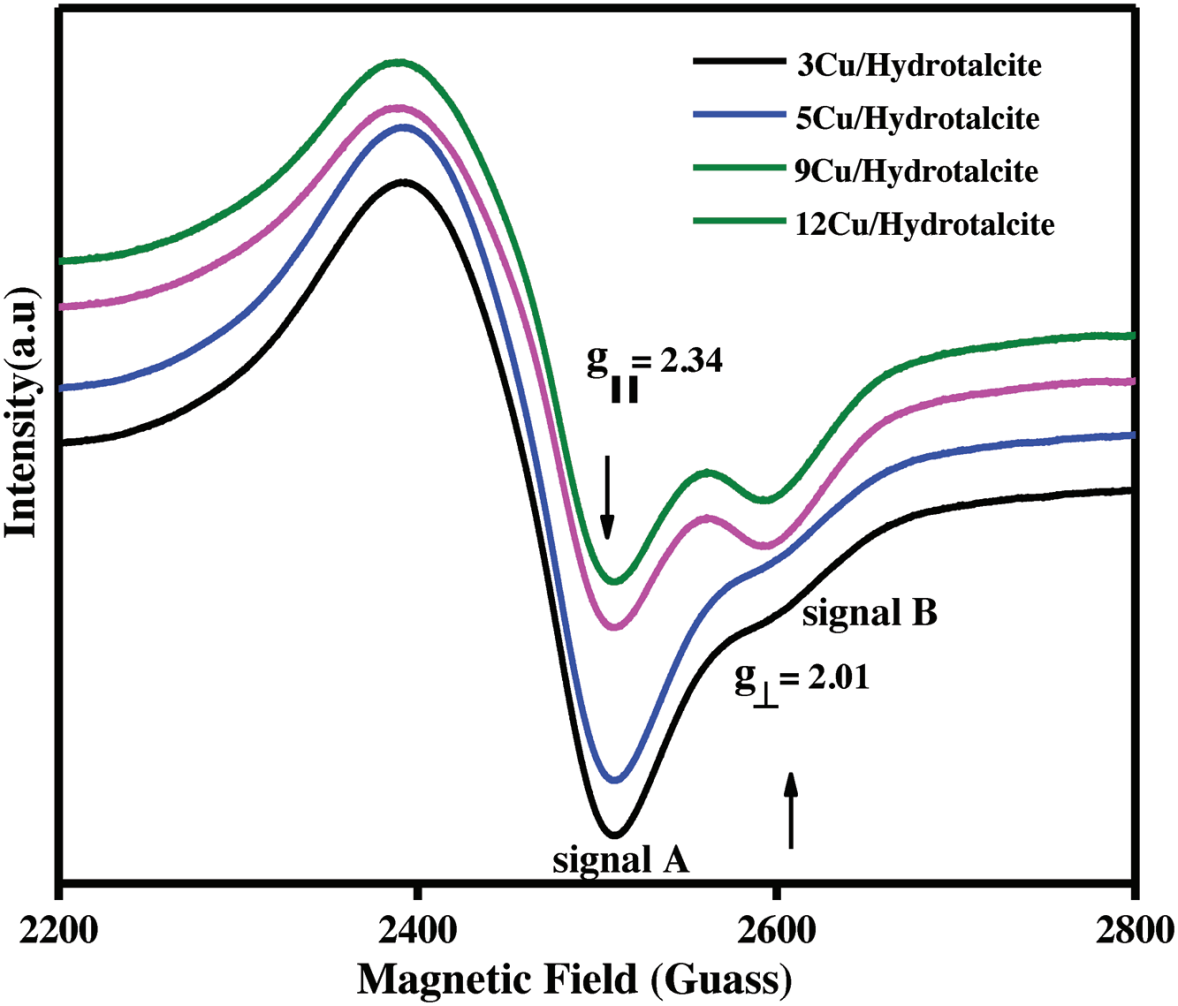
ESR patterns of various Cu‐loaded Hydrotalcite catalysts with g and A axial and perpendicular tensor parameters.

The electron paramagnetic resonance spectroscopy (EPR) is an important technique to investigate qualitative and quantitative analysis of isolated Cu^+2^ ions as well as coordination environment of isolated paramagnetic copper ions in the catalysts. The EPR parameters and spectra of all catalysts are presented in **Figure** **5** and Table S1 (Supporting Information). For all the samples, two kinds of EPR signals were observed; the first one is termed as signal A (Figure 5) that indicates the resolved hyperfine structure and the isolated Cu^+2^ ions, which are distorted and octahedrally coordinated.^33^, ^34^ The second one, signal B, shows an unresolved hyperfine splitting signal, which could be attributed to Cu^+2^ species interacting with each other. Generally, signals A, B are related to the clustered Cu^+2^ ions, with following g and A spin Hamiltonian hyperfine. And the tensor parameters g and A values, in axial and perpendicular for the Cu^+2^ ionic centers are g^⊥^ = 2.32, g^||^ = 2.01, A^||^ = 115G, and A^⊥^ = 133G respectively. Signal A and B tends to be broader with Cu loadings due to the formation Cu aggregates which lead to dipole–dipole magnetic interactions between adjacent Cu^+2^ ions (as bulk copper should be very close to each other). Yao et al.^35^ also delineated same for ZnO‐supported Cu catalysts.

**Figure 6:**
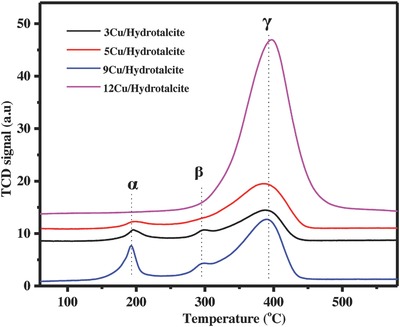
H_2_‐TPR profiles of various Cu loaded Hydrotalcite catalysts. Here, α, β, and γ are three different temperature regions.

Hydrogen‐temperature programmed reduction (H_2_‐TPR) profiles and the amount of hydrogen consumption of all copper loaded catalysts are shown in **Figure** **6**. All the samples displayed three types of reduction profiles in the range of 190–200 °C (denoted as α) and other two peaks between 270 and 425 °C (denoted as β and γ). Over the 3Cu/Hydrotalcite sample, a smaller reduction peak is appeared at ≈200 °C, i.e., a lower H_2_ consumption peak reduced to isolated Cu, which is neither Cu_2_O nor CuO species. The second broad peak at 270–388 °C attributes to higher H_2_ consumption region and the reduction of highly dispersed copper oxide species. The peak at ≈200 °C is ascribed to reduction of isolated Cu^2+^ ions (Cu^2+^ → Cu^+^), and the peak at ≈290 °C assigned to reduction of highly dispersed CuO (Cu^2+^ → Cu^+^ and Cu^+^ → Cu^0^). Consequently, the peak at ≈380 °C was attributed to the reduction of bulk Cu^+^ to Cu^0^,^36^, ^37^ respectively. At higher Cu loadings (i.e., ≥10 wt%), the reduction peak was shifted to a higher temperature region due to the formation of larger crystalline CuO particles, which are weakly interacted with the Hydrotalcite support, similar to the trend seen in our previous studies.^30^, ^32^ Thus, the reducibility behavior was confirmed by TPR profiles, and the results are also in very good agreement with XRD and electron spin resonance (ESR) analyses.

The total surface acidity properties of pure Hydrotalcite and various Cu‐loaded Hydrotalcite catalysts were analyzed by the NH_3_‐TPD (temperature programmed desorption) and are shown in Figure S1 (Supporting Information). Moreover, the acidity data of NH_3_ consumption values are presented in **Table** **3**. The NH_3_‐desorbed peaks were divided into three different temperature regions, i.e., the first region at 60–220 °C, second region at 200–450 °C, and third is above 450 °C; these correspond to weak, moderate, and strong acidic properties, respectively. Table 3 clearly indicates that the total acidity increased with the Cu incorporation due to the introduction of protonated sites in comparison with the pure Hydrotalcite support. But the total acidity decreased from 1.26 to 0.31 mmol g^−1^ with Cu loadings from 3 to 15 wt%. The declining trend of total acidity at higher copper loading may be due to the coverage of the larger CuO species, and this leads to low availability of acidic sites on the surface of these catalysts. The similar tendency is noticed by Pudi et al.^38^ and Yue et al.^39^ Probably, the moderate acidic sites of 3Cu/Hydrotalcite played an important role in condensation and dehydration steps and, thus, higher LA conversion was achieved.

**Table 3:** The acidity values of Hydrotalcite support and various Cu‐loaded Hydrotalcite catalysts

Cua) [wt%]	NH_3_ uptakea) [mmol g^−1^]		
	Weak	Moderate	Total acidity [mmol g^−1^]
0.0	0.04	0.32	0.36
3	0.08	1.18	1.26
5	0.09	0.78	0.87
9	0.11	0.41	0.52
12	0.12	0.31	0.43

^a)^ Measured from the temperature programmed desorption of NH_3_ analysis.

### 2.2 Activity Study

#### 2.2.1 Effect of Cu Loading on LA Hydrogenolysis

**Figure 7:**
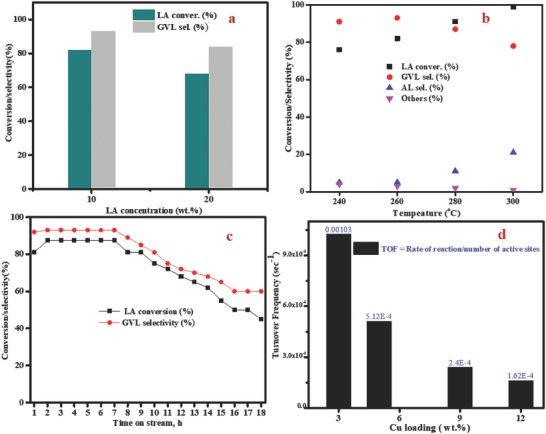
a) Effect of LA concentration. b) Effect of temperature. c) Time on stream (TOS) during on hydrogenolysis activity. d) The effect of Cu loading over turn over frequency (TOF) of various Hydrotalcite‐based Cu catalysts. Reaction conditions: 0.5 g of catalyst, reaction temperature = 260 °C, H_2_ flow rate = 30 mL min^−1,^ LA WHSV = 0.456 h^−1^. TOS = 23 h, LA = levulinic acid (LA), γ‐Valerolactone (GVL), Angelica lactones (ALs), and others include valeric acid (VLs), acetone, etc.

The activity results of vapor‐phase hydrogenolysis of LA to GVL were studied over various Cu/Hydrotalcite catalysts at 260 °C and 0.1 MPa H_2_ partial pressure, and results were presented in **Table** **4**. The pure Hydrotalcite showed very low LA conversion (≈4%) and the absence of GVL formation (Table 4), but only Angelica lactone (AL) was formed among all by‐products. Over 3Cu/Hydrotalcite catalyst, a maximum LA conversion of 87.5% and a GVL selectivity of 95% were attained, and the rest were the minor compounds such as valeric acid (VA) and AL. The declining trend in LA conversion from 87.5% to 56% and GVL selectivity from 95% to 67% was observed with Cu loadings from 3 to 15 wt%. Moreover, over 3Cu/Hydrotalcite catalyst was found to be superior in terms of GVL selectivity compared to Al_2_O_3_ and other zirconia‐based catalysts (Table 1) under specific reaction conditions. Chary and co‐workers^40^ reported less GVL selectivity of 87% for the Al_2_O_3_‐supported copper material. For LA hydrogenolysis, bifunctional properties are required; thus, as expected, Cu is a good hydrogenation catalyst. Moreover, the support Hydrotalcite provides the surface acidic properties, which play an important role in LA hydrogenolysis. In this study, the turnover frequencies (TOFs) were calculated by the reaction rates of LA as well as surface‐active Cu atoms as determined by the N_2_O titration method (**Figure** **7**d). As expected, 3Cu/Hydrotalcite catalyst possesses a higher TOF value of 1.03 × 10^−3^ s^−1^. Best activity results were obtained over 3 wt% Cu catalyst due to a higher number of ultrafine small particles that are narrowly distributed with an average size of ≈2 nm over Hydrotalcite surface, and, thus, high performance was achieved. In addition, the conversion of LA and selectivity decreased significantly with Cu loadings (from 3 to 12 wt%); this is due to the formation bigger particles, i.e., above ≈15 nm and also lower acidic sites.

**Table 4:** Activity data of LA hydrogenolysis using Hydrotalcite and various Cu‐loaded Hydrotalcite catalysts

Cua) [wt%]	*X*_LA_ [%]	Selectivity [%]			
		GVL	VA	AL	Others
0.0	4	0	0	10	90
3	87.5	95	4	1	0
5	73	83	5	3	11
9	62	77	3	12	8
12	56	67	1	18	14

^a)^ Reaction conditions: 0.5 g of catalyst, reaction temperature = 260 °C, pressure = 0.1 MPa H_2_; H_2_ flow rate = 30 mL min^−1^, WHSV = 0.456 h^−1^, LA: levulinic acid (LA), γ‐Valerolactone (GVL), Valaeric acid (VA), Angelica lactone (AL), and others. *X*_LA_ = LA conversion [%].

In the case of GVL production, a similar trend was observed with the TOF values, i.e., a decreasing trend with Cu loadings from 3 to 12 wt%. The TOF values were well correlated with the Cu particle size, and it has a negative effect on the LA hydrogenolysis activity. Over 3Cu/Hydrotalcite catalyst has the highest TOFs of GVL values, which were achieved due to high Cu dispersion (≈40%) in combination with higher acidity. Henceforth, the LA conversion and GVL selectivity directly correlated with the Cu particle size, dispersion, and also surface acidic properties of the catalyst. Lomate et al.^41^ also reported that Cu supported SiO_2_ appears to be a complex function of acidity, which is the nature of copper species in the Cu/SiO_2_ catalysts for LA hydrogenolysis. Zheng et al.^42^ and Zhu et al.^43^ performed hydrogenolysis reaction catalyzed by a copper‐supported material, correlation of surface‐active Cu species, and particle size versus activity. As demonstrated by Schittkowski et al.,^44^ their bifunctional nature of Cu/ZrO_2_ catalyst is applied in the hydrogenation of ethyl acetate. These distinct properties of copper‐supported catalysts reviewed for several parameters like the metal dispersion in correlation with the acid properties played an important role for LA hydrogenolysis.^45^, ^46^, ^47^

#### 2.2.2 Possible Reaction Mechanism for Hydrogenolysis of LA to GVL

**Scheme 1:**
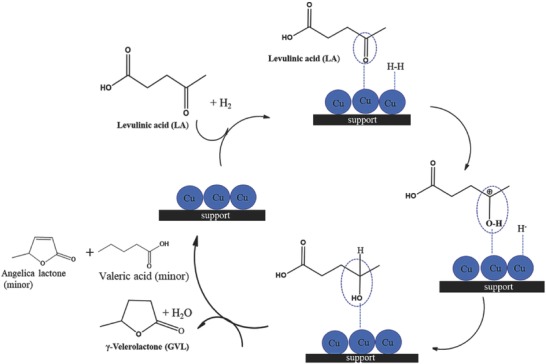
Possible mechanism for hydrogenolysis of LA to GVL over heterogeneous Cu/Hydrotalcite catalyst.

The proposed and plausible reaction mechanism for the hydrogenolysis of LA over Cu‐loaded Hydrotalcite catalysts was depicted in **Scheme** **1**. First, H_2_ and LA were chemisorbed on the Cu surface site followed by the heterolytic cleavage of the H—H bond takes place. One of the H atoms was transformed to the intermediate formation, which was stabilized by the interaction with the Cu surface sites. Then, second H atom participates in the formation of Cu‐bonded 4‐hydroxy valeric acid in the presence of acidic sites and, subsequently, promotes the formation of GVL. However, the synergic effect of acidic properties and the H_2_ spill‐over effect over Cu sites are crucial for the hydrogenolysis of LA. Above preliminary studies clearly demonstrated the promotional role of surface copper sites and acidic properties present in the network of the Cu/Hydrotalcite, where acid properties were created in the vicinity of metallic copper according to the proposed structure.^48^, ^49^ Through the characterization of NH_3_‐TPD and N_2_O decomposition techniques, both acidity and the amount of active Cu sites on Hydrotalcite were analyzed and reported in this study. Similar phenomena are reported in the previous studies.^50^, ^51^, ^52^

#### 2.2.3 Effect of Reaction Conditions

The reaction parameters such as LA concentration, reaction temperature, and stability test, i.e., time on stream (TOS) were performed on 3Cu/Hydrotalcite under 0.1 MPa H_2_ pressure. In Figure 7a, LA conversion drops from 87.5% to 68% and GVL selectivity from 95% to 84% with LA concentration from 10 to 20 w/w%, respectively. As expected, the decreased conversion is due to the insufficient number of active Cu sites on the catalyst surface. The most optimal LA concentration was found to be 10 wt/wt% in order to achieve maximum LA conversion with the highest GVL selectivity. Chary and co‐workers^40^ have also ascertained similar assumption in the hydrogenolysis of LA over Cu–ZrO_2_ catalyst.

The influence of reaction temperature was also studied in similar reaction conditions using the same catalyst (Figure 7b). At 240 °C, the LA conversion is around 76% which was lower than that at this high reaction temperature. As expected, the temperature had a significant effect on LA conversion.^53^ The LA conversion increased from 87.5% to 99% with reaction temperature from 260 to 300 °C. Moreover, the selectivity toward the GVL decreased from 95% to 75% and increased the formation of by‐products such as VA and AL. According to the thermodynamic analysis, at higher temperature (>260 °C), dehydration of LA and also ring opening of GVL are favored.^54^

The TOS, i.e., stability of 3Cu/Hydrotalcite catalyst was investigated at 260 °C under atmospheric pressure (Figure 7c). Initially, during 1–8 h TOS, the LA conversion increased from 81% to 87.5% with a constant GVL selectivity of 95%. After 8 h, the conversion gradually decreases and attained ≈55% at 23 h. It shows that the catalyst was stable up to 8 h; after that a drop in conversion and selectivity occurs. The declining trend was observed due to catalyst deactivation by coke deposition on the surface.

#### 2.2.4 Activity Study of Spent Catalyst

**Figure 8:**
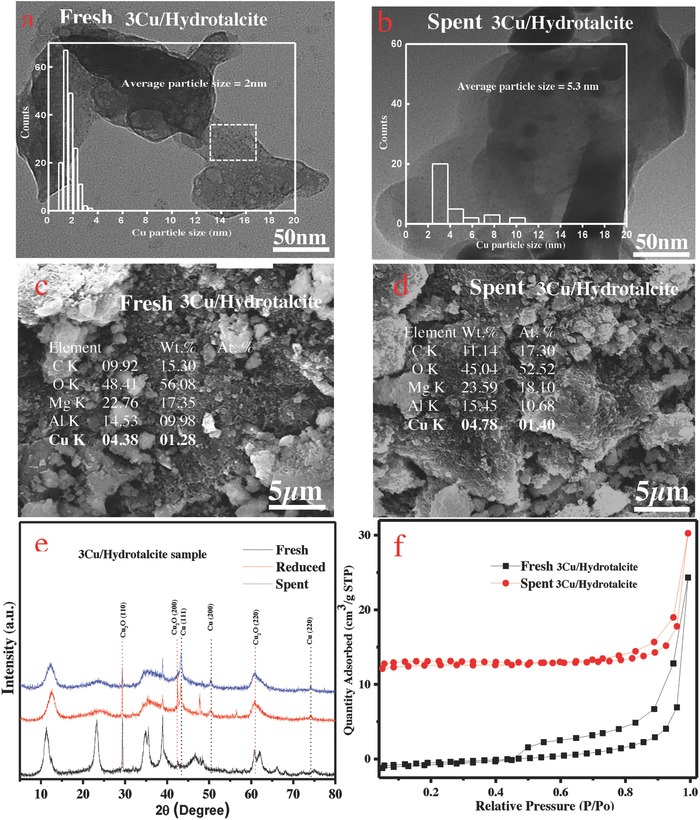
Characterization of the fresh and spent 3Cu/Hydrotalcite after one cycle: a,b) TEM images; c,d) SEM images; e) XRD patterns; and f) N_2_ adsorption–desorption.

In order to understand the spent catalyst activity, structural aspects of fresh and spent 3Cu/Hydrotalcite catalyst have to be analyzed. The LA hydrogenolysis reaction was carried out under similar reaction conditions (**Table** **5**). It was observed that the spent catalyst has shown 79% LA conversion and 71% GVL selectivity, respectively. Thus, the decrease in activity is confirmed by the structural characterizations such as TEM, SEM, XRD, N_2_ physisorption, and NH_3_‐TPD analysis (**Figure** **8** and Table 5).

**Table 5:** Physico‐chemical properties and activity study of fresh and used catalyst of 3Cu/Hydrotalcite

3Cu/Hydrotalcitea)	BET surface areab) [m^2^ g^−1^]	Total NH_3_ uptakec) [mmol g^−1^]	Pore volumeb) [cc g^−1^]	Pore‐diameterb) [nm]	Particle size d) [nm]	*X*_LA_ Conv. [%]	GVL sel. [%]
Fresh	119	1.26	0.040	30.2	2.1	87.5	95
Spent	70	0.62	0.028	33.4	5.3	71	79

^a)^ Reaction conditions: 0.5 g of catalyst, reaction temperature = 260 °C, 0.1 MPa H_2_; H_2_ flow rate = 30 mL min^−1^, LA WHSV = 0.456 h^−1^, γ‐valerolactone (GVL)

^b)^ Surface area measured from nitrogen adsorption–desorption isotherm

^c)^ Measured from the TPD of NH_3_

^d)^ ‐Determined by TEM analysis.

TEM images and the particle size distribution of spent 3Cu/Hydrotalcite catalysts were displayed in Figure 8a,b. After the reaction, the average Cu particle sizes increased from 2 to 5.3 nm due to the agglomeration of Cu nanoparticles during the course of the reaction which influenced the LA conversion. The SEM–energy dispersive X‐ray (EDX) results of the fresh and spent samples are shown in Figure 8c,d. From the XRD results, the spent catalyst 3Cu/Hydrotalcite had peaks at 2θ = 34.1°, 38.1° 77.1°, 44.2 °, and 50.1 °, which correspond to Cu (+1), Cu (0), respectively (Figure 8e). Henceforth, the active Cu sites in spent catalysts transformed to inactive Cu oxides due to change in oxidation state. In addition, BET surface area and acidity–basicity of the spent catalyst were also decreased (Table 5). Thus, the catalyst deactivation was confirmed by the aforementioned analysis due to decline in key properties of the catalyst.

## 3 Conclusions

In summary, transformation of biomass‐based LA is a sustainable route to produce GVL. In this study, various Cu over HT ‐based catalysts are prepared with 3–12 wt% Cu loadings. The role of support and the Cu active sites played an important role as well tandem bifunctionality of Cu/Hydrotalcite catalysts. The formation of a Hydrotalcite structure between the interlayered carbonate anions, which are responsible for the redox character and acidity properties. Cu loadings had a significant effect on the physicochemical properties of the catalysts in achieving superior performance at lower loading, i.e., 3 wt%. Cu found to be the most suitable catalyst for the LA hydrogenolysis due to its enhanced activity. Fresh and spent catalysts were characterized to understand the stability and structural changes occurred in the catalysts. In the future, Cu‐based bimetallic and mixed oxide catalysts will be developed to improve the catalyst durability.

## 4 Experimental Section

*Preparation of Cu/Hydrotalcite Catalyst—Synthesis of Hydrotalcite Support*: In a typical procedure, Hydrotalcite was prepared by co‐precipitation.^55^ For this purpose, a solution containing Mg(NO_3_)_2_ · 6H_2_O (≈1 mole) and Al (NO_3_)_3_ · 9 H_2_O (≈ 0.5 moles) in a 700 mL distilled water, denoted as solution A, was prepared, while solution B was prepared by dissolving 50% aqueous NaOH (≈3.5 moles) and anhydrous Na_2_CO_3_ (≈0.943 moles) in a 1000 mL distilled water. Furthermore, solution A was added to solution B dropwise and mixed under vigorous stirring of a ramp rate of 2 mL min^−1^ for 18 h at 60 °C in a hot bath. Then, the final white precipitate was filtered off and washed several times with hot deionized water until neutral pH was achieved. The resulted white precipitate material was dried under vacuum at 50 °C overnight, and then calcined at 450 °C for 5 h under air and denoted as a Hydrotalcite.

*Preparation of Cu/Hydrotalcite Catalyst—Synthesis of Cu Colloidal Solution*: In the beginning, Cu colloids with sizes in the range of 2–18.3 nm were synthesized by the thermal decomposition of copper acetate in tri‐octylamine and oleic acid at optimal temperatures and with the aid of surfactants.^56^ The copper colloidal size could be tuned by controlling the synthesis parameters such as molar ratio of copper (I) acetate to oleic acid and the exact size was determined by TEM analysis, which are shown in Figure S2 (Supporting Information).

*Depositing Cu on Hydrotalcite Support—Colloidal‐Deposition Method*: Cu/Hydrotalcite catalysts with the various Cu loading (3–12 wt%) but with different Cu particle sizes were prepared by a colloidal‐deposition method; here ethanol was used as a solvent. Requisite amounts of Hydrotalcite and copper colloid solution were dissolved in ethanol, and the suspension was stirred at 70–80 °C for 4 h. The solid was collected by filtration, followed by drying at 50 °C overnight under vacuum, and calcination at 300 °C in air for 5 h. This results in Cu/Hydrotalcite samples (following TEM images, see in Figure 3) followed by vacuum drying at 50 °C overnight and calcination at 300 °C in air for 5 h.

XRD patterns were measured with a Rigaku Miniflex diffractometer using Cu Kα radiation (λ = 1.5406 Å) at 40 kV and 30 mA. The average metal crystallite size was calculated using the Scherrer equation. The exact amount of Cu loadings was measured by inductively coupled plasma atomic emission spectroscopy (ICP‐AES). The SEM analysis of the samples was carried out by Hitachi S‐520 SEM unit, equipped with an EDS. TEM analysis was performed with a JEOL 2010 electron microscope operating at 200 kV. The textural properties that include specific surface area, pore diameter, and pore volume analysis of the catalysts were analyzed by N_2_ physisorption at −197 °C using Quanta chrome Autosorb‐II instrument using the BET method. EPR of the pre‐calcined samples was recorded on a Bruker EMX‐X band spectrometer at the X‐band frequency of 9.7667 GHz at room temperature.

*Hydrogen‐Temperature Programmed Reduction*: Reducibility of the catalysts was measured using H_2_ temperature programmed reduction on Micromeritics 2920 instrument. Briefly, about 0.2 g of the sample was loaded in a U‐shaped sample tube. Prior to analysis, the sample was heated at 120 °C for 2 h in helium flow to remove moisture and then cooled to room temperature. Reduction was carried out using 5% H_2_/Ar with a flow rate of 50 mL min^−1^ from room temperature to 650 °C with a heating rate of 5 °C min^−1^. Amount of H_2_ consumed and *T*_max_ were calculated using the GRAMS/32 software.

*N_2_O Titration Analyses*: Metallic Cu was oxidized to Cu_2_O by decomposition of N_2_O. The number of copper atoms on the surface was calculated using molar stoichiometry of N_2_O/Cu = 0.5. Copper metal area, particle size, and dispersion were measured based on refs. 32, 57. Copper metal surface area was calculated assuming an atom density of 1.47 × 10^19^ Cu atoms m^−2^. Dispersion is defined as the ratio of the number of surface Cu atoms to the total number of Cu atoms in the catalyst. Average particle size of Cu was calculated using Equation (1).

(1) $$D_{\mathrm{Cu}}\;=\frac{A\;\times \;S\;\times \;M_{\mathrm{Cu}}}{m_{\mathrm{cat}}.w_{\mathrm{Cu}}\;\left(\mathrm{wt}\%\right)}\;\;\times \;100$$

where *A* corresponds to the amount of H_2_ consumption (mL g^−1^) from the integrating the area of TCD signals (in terms of moles), *S* is the stoichiometric factor, in this case, *S* = 2 for Cu; *M*_Cu_ is the atomic molecular weight of Cu (63.546 g mol^−1^), *m*_cat_ is the mass of the catalyst (g), and *w*_Cu_ is the mass fraction of Cu in the catalyst.

*Temperature Programmed Desorption (NH_3_ TPD)*: The total acidity of the catalysts was analyzed by NH_3_‐temperature programmed desorption (NH_3_‐TPD) in a Micromeritics 2920 instrument. Prior to analysis, 0.2 g of sample was placed in a U‐shaped sample tube and heated at 300 °C for 2 h in helium flow and then cooled to 60 °C. Ammonia balanced helium was passed over the sample with a flow rate of 40 mL min^−1^ for 1 h until saturation was obtained; then mixture gas was switched to helium to remove physisorbed ammonia gas. The temperature was increased to 800 °C with a heating rate of 10 °C min^−1^ to get an ammonia signal. The amount of ammonia desorbed was measured using GRAMS/32 software.

*Catalytic Activity Test*: Vapor‐phase LA hydrogenolysis reaction was investigated in a fixed‐bed reactor operating at 0.1 MPa H_2_ pressure. In a typical activity run, 0.5 g of catalyst (40–60 µm mesh size) diluted with an equal amount of quartz grains was used. Prior to the activity test, the catalyst was reduced in situ at 250 °C for 3 h under 5% H_2_–Ar flow (30 mL min^−1^). In the next reduction, the reactor was cool down to the reaction temperature. Aqueous LA (10 wt%) feed was introduced into the reactor by high performance liquid chromatography (HPLC) pump, along with the premixed H_2_ at a flow rate of 30 mL min^−1^ (constant weight hour space velocity (WHSV) = 0.456 h^−1^). The performance of the catalysts was investigated at different operating and reaction conditions such as temperature, and H_2,_ and LA concentrations. The liquid and gaseous products were collected and analyzed in gas chromatography (GC) every hour, equipped with a ZB‐5 capillary (Agilent) column via a flame ionization detector (FID). The major products identified during the LA hydrogenolysis were GVL, AL, and VL. The conversion of LA (Equation (2)) and the selectivity (Equation (3)) was calculated according to the following equations

(2) $$\mathrm{LA}\;\mathrm{conversion}\;\;\left(\%\right)=\frac{{\left[\mathrm{moles}\;\mathrm{of}\;\mathrm{LA}\right]}_{\mathrm{in}}\;-\;{\left[\mathrm{moles}\;\mathrm{of}\;\mathrm{LA}\right]}_{\mathrm{out}}}{{\left[\mathrm{moles}\;\mathrm{of}\;\mathrm{LA}\right]}_{\mathrm{in}}\;}\;\;\times \;100$$

(3) $$\mathrm{GVL}\;\mathrm{selectivity}\;\;\left(\%\right)=\frac{{\left[\mathrm{moles}\;\mathrm{of}\;\mathrm{GVL}\right]}_{\mathrm{out}}}{{\left[\mathrm{moles}\;\mathrm{of}\;\mathrm{GVL}\right]}_{\mathrm{in}}\;-\;{\left[\mathrm{moles}\;\mathrm{of}\;\mathrm{GVL}\right]}_{\mathrm{out}}}\;\;\times \;100$$

The carbon balance in all the activity tests found to be ≥96%, and the error of ±4% was found and this is under the acceptable limits.

## Conflict of Interest

The authors declare no conflict of interest.

## Supporting information

Supplementary
