# Supplementary material for: Efficient Vapor‐Phase Selective Hydrogenolysis of Bio‐Levulinic Acid to γ‐Valerolactone Using Cu Supported on Hydrotalcite Catalysts
Source: Glob Chall. 2018 Jul 11;2(12):1800028. doi: 10.1002/gch2.201800028 (PMC6360448; doi:10.1002/gch2.201800028)
Supplement: Supplementary file 1 — Supplementary [file GCH2-2-1800028-s001.pdf]

Copyright WILEY-VCH Verlag GmbH & Co. KGaA, 69469 Weinheim, Germany, 2018.

Global Challenges
Open Access

Supporting Information

for *Global Challenges,* DOI: 10.1002/gch2.201800028

Efficient Vapor-Phase Selective Hydrogenolysis of Bio-Levulinic Acid to γ-Valerolactone Using Cu Supported on Hydrotalcite Catalysts

Harisekhar Mitta, Prem Kumar Seelam, K. V. Raghava Chary, Suresh Mutyala, Rajender Boddula, Inamuddin,* and Abdullah M. Asiri**

Supporting information

Table S1 Characteristics of the electron paramagnetic resonance spectroscopy patterns observed in various Cu loaded Hydrotalcite catalysts

Cu loading (wt.%)	$g^{\parallel}$	$A^{\parallel}$ (G)	$g^{\perp}$	$A^{\perp}$ (G)
3	2.33	115	2.01	126
5	2.36	114	2.02	123
9	2.36	113	2.03	120
12	2.36	110	2.03	120

Note: Spin Hamiltonian tensor parameters in axial and perpendicular g ($g^{\parallel}$ and $g^{\perp}$) and A ($A^{\parallel}$ and $A^{\perp}$ (G) for theCu^{+2} Centers in the various hydrotalcite catalysts

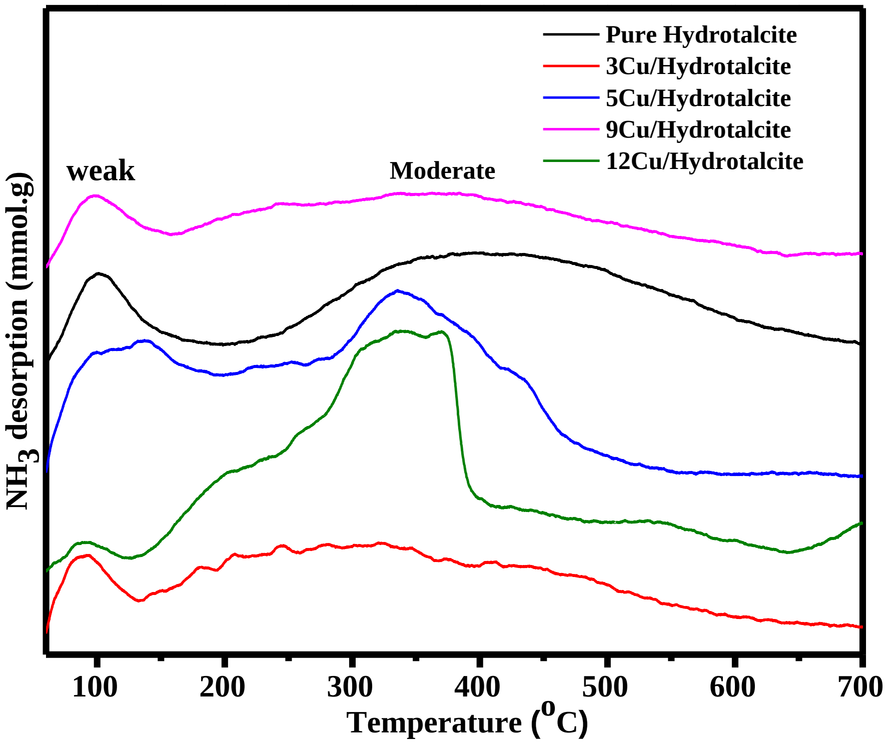

Figure S1. NH_3-temperature programmed desorption profiles of pure Hydrotalcite support and various Cu loaded Hydrotalcite catalysts.

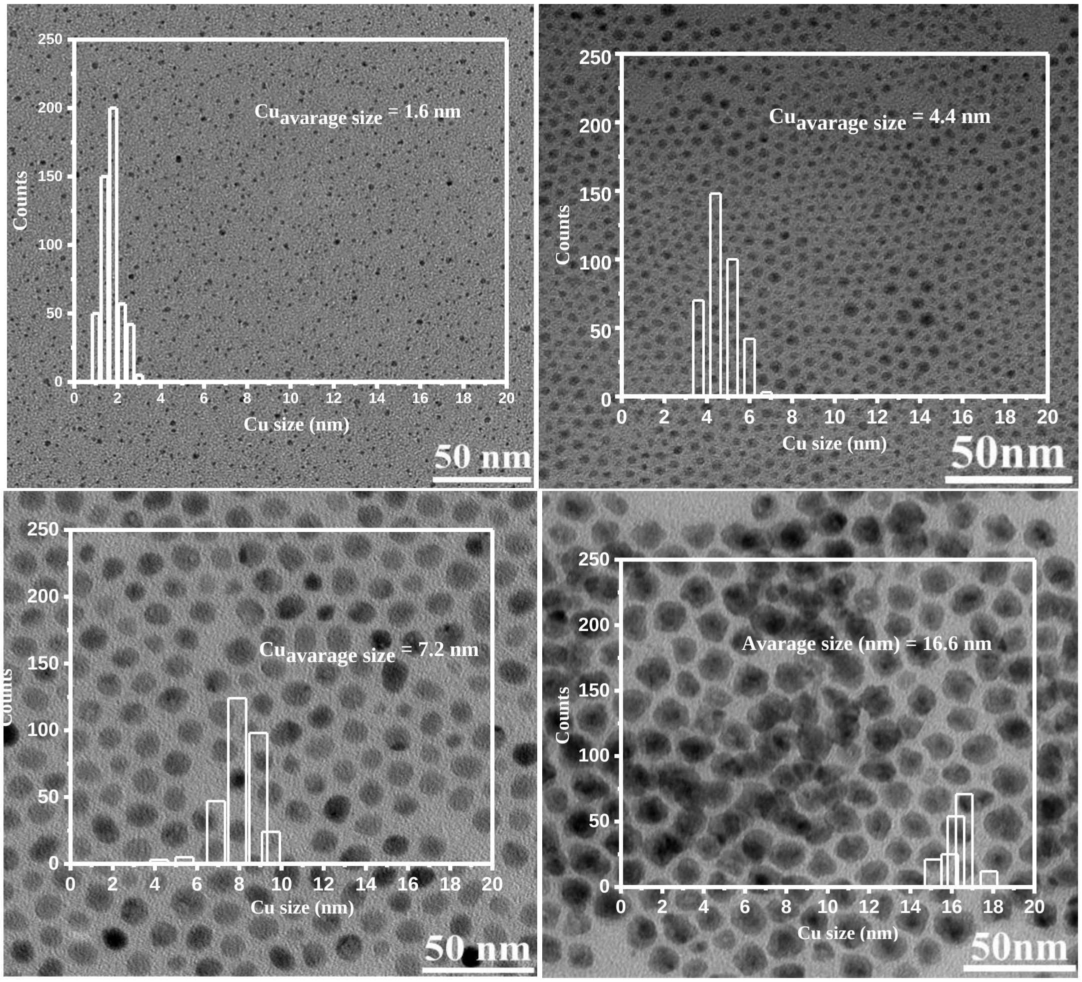

Figure S2. The particle distributions and TEM images of Cu colloids with diameters of ~2 to ~16.6 nm.
